# Development of RT-qPCR and semi-nested RT-PCR assays for molecular diagnosis of hantavirus pulmonary syndrome

**DOI:** 10.1371/journal.pntd.0007884

**Published:** 2019-12-26

**Authors:** Bruno Tardelli Diniz Nunes, Maria Helena Rodrigues de Mendonça, Darlene de Brito Simith, Adriana Freitas Moraes, Carla Conceição Cardoso, Ivy Tsuya Essashika Prazeres, Ana Alice de Aquino, Alessandra da Conceição Miranda Santos, Alice Louize Nunes Queiroz, Daniela Sueli Guerreiro Rodrigues, Regis Bruni Andriolo, Elizabeth Salbé Travassos da Rosa, Livia Carício Martins, Pedro Fernando da Costa Vasconcelos, Daniele Barbosa de Almeida Medeiros

**Affiliations:** 1 Department of Arbovirology and Hemorrhagic Fevers, Evandro Chagas Institute, Ananindeua, Brazil; 2 Post-Graduation Program in Virology, Evandro Chagas Institute, Ananindeua, Brazil; 3 Department of Community Health, Pará Estate University, Belém, Brazil; Naval Medical Research Center; Biological Defense Research Directorate, UNITED STATES

## Abstract

Hantavirus Pulmonary Syndrome is an, often fatal, emerging zoonotic disease in the Americas caused by hantaviruses (family: *Hantaviridae*). In Brazil, hantavirus routine diagnosis is based on serology (IgM-ELISA) while RT-PCR is often used to confirm acute infection. A Semi-nested RT-PCR and an internally controlled RT-qPCR assays were developed for detection and quantification of four hantaviruses strains circulating in the Brazilian Amazon: Anajatuba (ANAJV) and Castelo dos Sonhos (CASV) strains of Andes virus (ANDV) species; and Rio Mamoré (RIOMV) and Laguna Negra (LNV) strains of LNV species. A consensus region in the N gene of these hantaviruses was used to design the primer sets and a hydrolysis probe. *In vitro* transcribed RNA was diluted in standards with known concentration. MS2 bacteriophage RNA was detected together with hantavirus RNA as an exogenous control in a duplex reaction. RT-qPCR efficiency was around 100% and the limit of detection was 0.9 copies/μL of RNA for RT-qPCR and 10 copies/μL of RNA for Semi-nested RT-PCR. There was no amplification of either negative samples or samples positive to other pathogens. To assess the protocol for clinical sensitivity, specificity and general accuracy values, both assays were used to test two groups of samples: one comprising patients with disease (n = 50) and other containing samples from healthy individuals (n = 50), according to IgM-ELISA results. A third group of samples (n = 27) infected with other pathogens were tested for specificity analysis. RT-qPCR was more sensitive than semi-nested RT-PCR, being able to detect three samples undetected by conventional RT-PCR. RT-qPCR clinical sensitivity, specificity and general accuracy values were 92.5%, 100% and 97.63%, respectively. Thus, the assays developed in this study were able to detect the four Brazilian Amazon hantaviruses with good specificity and sensitivity, and may become powerful tools in diagnostic, surveillance and research applications of these and possibly other hantaviruses.

## Author summary

Hantavirus Pulmonary Syndrome (HPS) is a serious and often fatal disease caused by viruses known as hantaviruses. These viruses are harbored by wild rodents and people can become infected through contact with infected-rodents droppings, urine or saliva. After an incubation time of 1–8 weeks, patients usually present flu-like symptoms such as fever, fatigue and muscle aches, although some patients may also present headaches, dizziness, chills, nausea, vomiting, diarrhea, and abdominal pain. It is only 4–10 days after initial symptoms, however, that the severe stage of disease takes place. Symptoms include coughing, shortness of breath and eventually the lungs fill with fluid which can lead to shock and death. As such, HPS should be diagnosed quickly as any delay may have great impact on patient recovery. However, given the unspecific nature of early symptoms, clinical diagnosis of HPS is difficult and laboratory assays are needed to confirm hantavirus infection as soon as possible, helping physicians to choose the most adequate treatment. In this study, we developed new laboratory assays that can help detect the virus in infected patients in early stages of disease. In addition, we showed these assays have a good performance in discriminating HPS from other similar diseases by testing not only several samples collected from both HPS patients and healthy individuals but also samples infected with other pathogens. Our results show that these assays may become important tools for rapid, sensitive and specific diagnosis of HPS.

## Introduction

Hantaviruses (order *Bunyavirales*, family *Hantaviridae*, genus *Orthohantavirus*)[1–3] are etiologic agents of anthropozoonotic diseases comprising of two clinical entities: Hemorrhagic Fever with Renal Syndrome (HFRS), which occurs in the Old World, and Hantavirus Pulmonary Syndrome (HPS) in the New World[4]. Hantavirus life cycle involves transmission between small wild mammals, specially rodents (*Cricetidae* family, subfamilies *Sigmodontinae* and *Arvicolinae* in the Americas)[5–7] but also shrews, moles[8] and bats[9–11]. Humans are accidental hosts and transmission occurs through inhalation of aerosols produced from excreta of infected rodents[12–14]. Hantavirus are enveloped viruses with tri-segmented negative sense RNA genome. The genome segments are named according to their size: L (Large), M (Medium) and S (Small)[15,16].

In Brazil, the first confirmed cases were notified in 1993 and since then 2080 cases were recorded with 833 deaths. The Amazon region includes 20 million people living in about 60% of Brazilian territory and covers approximately one third of the total area of South America. The region contain a wide diversity of *Sigmodontinae* and *Cricetidae* rodents[17]. The first record of HPS in the Brazilian Amazon occurred in 1995 in Castelo dos Sonhos district in Altamira city, Para State[18]. After a long period of epidemiological silence, an outbreak occurred in 2000 in the municipality of Anajatuba, Maranhão State[19]. Later, several other outbreaks and isolated cases of HPS have been reported in the states of Amazonas, Maranhão, Mato Grosso, Pará and Rondônia. Two hantavirus species have been detected in the Amazon region. *Laguna Negra orthohantavirus* (LNV), which is commonly found in northern and southeastern Mato Grosso, was identified both in humans and in *Calomys callidus* rodents[20,21]. Furthermore, LNV also includes Rio Mamoré virus (RIOMV), according to the new classification by ICTV[1]. The first HPS case caused by RIOMV in Brazil was identified in the Amazon region[22] as well as its detection in *O*. *microtis* rodents captured in Amazonas and Rondonia states[23]. The other hantavirus species is *Andes orthohantavirus* (ANDV) which includes two closely related hantavirus strains: Anajatuba (ANAJV), and Castelo dos Sonhos (CASV)[23,24]. CASV has an high incidence in Pará state and had one detection in Mato Grosso State, in the region along the BR-163 highway linking Cuiaba to Santarém cities[25]. A single case of HPS caused by CASV was registered in Amazonas[23]. ANJV is associated with HPS cases in Maranhão state and was also identified in *O*. *aff*. *Fornesi* rodents[26].

Laboratory diagnosis of HPS is often conducted by Enzyme-Linked Immunosorbent Assay (ELISA) for detection of IgG and IgM specific antibodies in serum or blood of suspected patients. Given that IgM antibodies are detectable early in the disease, IgM ELISA is considered the reference method for HPS diagnostics. ELISA is also used to detect IgG antibodies in blood samples collected from rodents captured in eco-epidemiological investigations performed to estimate rodents infection rate[27–29]. In addition, immunohistochemistry can be used to detect viral antigens in tissues and viral RNA can be detected through RT-PCR in blood/serum and tissues samples. Most hantavirus RT-PCR protocols use nested format[30–35]. This strategy has provided a considerable increase in RT-PCR sensitivity, as shown before[33], despite also posing a higher risk of cross contamination.

Quantitative RT-PCR (RT-qPCR) is another molecular assay widely used to detect and quantify hantavirus genome, with a series of advantages over conventional RT-PCR[36–39]. Most of these assays, however, targets Old World hantaviruses[24,40–48], whereas some targets ANDV[49] and *Sin Nombre orthohantavirus* (SNV)[36,39,50,51]. Two RT-qPCR assays were developed to detect Juquitiba (JUQV), Araraquara (ARAV), RIOMV and possibly other hantaviruses circulating in South America[52,53].Although IgM ELISA is the reference assay for HPS diagnostic, it has some pitfalls such as: 1) the need to investigate seroconversion by testing a new sample collected two to three weeks after the first one, when the first serum is considered inconclusive, and 2) false-positive results due antigen cross-reactivity[54]. The addition of a molecular assay for virus genome detection in HPS diagnostics may be of help to overcome this problem. In this respect, we aimed to provide new molecular assays for detection of hantavirus genome, by developing an internally controlled RT-qPCR assay as well as a Semi-Nested RT-PCR protocol for detection of hantaviruses circulating in the Amazon region. These assays could be used as an add-on diagnostic tool in conjunction with IgM ELISA. We expect these new molecular assays to present higher clinical specificity values than commonly used serological assays such as ELISA, due to the lack of cross-reactivity observed in these assays, with comparable clinical sensitivity values.

## Methods

### Primer and probe design

Primers and probes were designed based on a sequence alignment of N gene, S Segment, from orthohantaviruses circulating in Brazilian Amazon using Geneious Pro program—R7 version (Biomatters, Auckland, New Zealand). A conserved region in the initial third of N gene was used to design two primers and a FAM labeled dual quenched probe for RT-qPCR, and three primers for semi-nested RT-PCR. Two primers (MS2F and MS2R) and a HEX labeled probe (MS2s) were used to detect MS2 bacteriophage genomic RNA (Roche Diagnostics, Risch-Rotkreuz, Switzerland) as a non-competitive exogenous internal control (EIC)[55]. Primers and probes were synthesized by Integrated DNA Technology (IDT). All primers and probes sequences used in this study are listed in Table 1.

**Table 1:** Nucleotide sequences of primers and probes used in hantavirus RT-qPCR and Nested RT-PCR assays.

Primer/Probe	Sequence (5'-3')	Position	Amplicon
HTNgen145F	GCAGCTGTGTCTACATTGGAGAA	145–167	90 bp
HTNgen234R	TGGTTTTGAAGCCAGTTTTTGA	213–234	
HTNgen169p	**FAM** AAACTC/**ZEN**/GCAGAACTCAAGAGA CAGCTGGC **IwBFQ**	169–197	
MS2 F	CATAAGTTAGATGGCCGTCTGT	841–863	123 bp
MS2 R	TAGAGACGACAACCATGCCAAAC	941–964	
MS2 probe	**VIC** TCCAGACAACGTGCAACATATCGCGACGTATCGTGATATGG **BHQ2**	881–992	
HTN_73F	CT**H**AAAGATGCCGAGAAGGCA	73–93	890 bp
HTN_963R	AACATAAAGTGCAGTTGG**D**GG	943–963	
HTN_314R	TTGACATC**H**AGGACATTCCCA	294–314	241 bp

### RNA extraction

RNA from serum or blood samples were extracted using 250μL of sample spiked prior to extraction with 5μL of EIC containing 20ρg/μL of MS2 RNA. The extractions were performed using TRIzol plus RNA Purification kit (Ambion, Thermo-Fisher Scientific, Waltham, USA) according to manufacturer's instructions, except that RNA was eluted with RNA Storage Solution (Applied Biosystems, Thermo-Fisher Scientific, Waltham, USA) instead of water.

### Plasmid cloning and RNA in vitro transcription

A double-stranded DNA fragment with 500bp comprising a sequence from a consensus region of hantavirus N gene was commercially synthesized (IDT, Iowa, USA). This fragment was cloned into a plasmid vector (PGEM-T Easy Vector System–Promega, Madison, USA) according to manufacturer’s instructions. Bacterial strain DH10B (Thermo-Fisher Scientific, Waltham, USA) was used as the *E*. *coli* host for construction and propagation of cDNA clones. Plasmid extraction was performed using Pure Link Quick Plasmid Miniprep kit (Invitrogen, Thermo-Fisher Scientific, Waltham, USA) according to manufacturer’s instructions. Presence of virus-specific sequence in clones was verified by restriction enzyme digestion with EcoRI (New England Biolabs, Massachusetts, EUA) and validated by Sanger DNA sequencing in a ABI 3130 automated sequencer (Applied Biotechnologies, Thermo-Fisher Scientific, Waltham, USA). To obtain virus-specific negative sense RNA, plasmids were in vitro transcribed using MegaScript SP6 kit (Ambion, Thermo-Fisher Scientific, Waltham, USA) following manufacturer’s protocol. Next, in vitro transcribed RNA was purified using MegaClear kit (Ambion, Thermo-Fisher Scientific, Waltham, USA), and eluted with RNA storage solution (Ambion, Thermo-Fisher Scientific, Waltham, USA) after precipitation. RNA quantification was performed in a Qubit digital fluorimeter using Qubit RNA BR Assay (Invitrogen, Thermo-Fisher Scientific, Waltham, USA) and results expressed in ƞg/μl. RNA copy number was then determined using the following formulae: C x A/L, whereas C is the amount of RNA expressed in g/mL; A represents Avogadro constant; and L is the RNA length in nucleotides versus 340, which corresponds to the medium molecular weight of a RNA nucleotide in g/mol. Conversion to genome copies per mL was made by using the following formulae: C x R x 1000/S, whereas C is the RNA concentration in copies/μL, R is the RNA elution volume (μL) and S is the sample volume (μL) used in RNA extraction.

### RT-qPCR Reactions

Hantavirus RT-qPCR reactions were performed in two steps. First, reverse transcription was carried out using 10μL of RNA mixed with 4 μL of Superscript VILO Master Mix (Invitrogen, Thermo-Fisher Scientific, Waltham, USA) containing SuperScript^™^ III RT, RNaseOUT^™^ recombinant ribonuclease inhibitor, random primers, MgCl2, dNTPs, and nuclease free water up to 20μL final reaction volume. This reaction mixture was incubated at 25ºC for 10 minutes, then for 1 hour at 42ºC and finally for 5 minutes at 85ºC for termination. In the second step, cDNA obtained in the previous step was used as template for qPCR. The qPCR reactions were carried out using TaqManUniversal Master Mix II with UNG kit (Applied Biosystems, Thermo-Fisher Scientific, Waltham, USA) containing 2 μL of cDNA; 0.5μL of each HTNgen145F and HTNgen263R primers (final concentration of 900nM) and 0.5μl of HTNgen169s probe (final concentration of 250nM) in a 10 μL final volume reaction. For each sample reactions were performed in triplicate in ViiA7 qPCR System (Applied Biosystems, Thermo-Fisher Scientific, Waltham, USA) using the following cycling conditions: 2 minutes at 50ºC and 15 minutes at 95ºC followed by 45 cycles of 15 seconds at 95ºC and 1 minute at 60ºC. Duplex reactions with MS2 EIC were first evaluated by comparing orthohantavirus genome amplification performance in singleplex and in duplex reactions using equimolar concentrations of both orthohantavirus and MS2 primers/probe sets. As expected, some competition was observed during amplification of both targets in the same reaction, thus the duplex protocol was further optimized by testing five different MS2 primer concentrations (from 700 to 50 nM) and four different MS2 probe concentrations (from 200–50 nM). The goal was to achieve the limiting primer/probe concentration for MS2 detection so that it could not interfere with target amplification. Only samples with CT values < 40 in two or more replicates were considered positive[56]. Samples with indeterminate CT values or with CT >40 in at least two replicates were considered negative.

### Semi-nested RT-PCR reactions

The semi-nested RT-PCR protocol used here was adapted from Johnson et al (1997) by replacing the primers with those designed in this study. Three degenerated primers, two externals (HTN_73F and HTN_963R) and one internal (HTN_314R), were used to amplify hantavirus genome in two rounds of PCR amplification after reverse transcription. RT reactions were carried out as described above for the RT-qPCR. For the first round of PCR amplification, 5 μL of cDNA was mixed with 45 μL of a mixture containing PCR buffer (250mM Tris-HCl pH 8.3, 100mM NaCl, and 0.1mM EDTA), magnesium chloride (1.5mM), dNTPs (0.2mM), 1μM of each HTN_73F and HTN 963R primers and 2.5U of Platinum Taq Polymerase (Invitrogen, Thermo-Fisher Scientific, Waltham, USA). Cycling conditions used were as follows: initial denaturation step at 95ºC for 1 minute followed by 35 cycles of 95ºC for 30 seconds, 55°C for 1 minute and 72ºC for 2 minutes, and a final extension step at 72ºC for 10 minutes. The second round of PCR amplification was carried out using 5μl of PCR product from the previous step diluted 100x and using the same reaction composition and cycling conditions from the first round, except by replacing the reverse primer for the internal HTN_314R primer. The PCR products from the final round of amplification were submitted to agarose gel electrophoresis and stained with SYBR safe gel stain (Molecular Probes, Thermo-Fisher Scientific, Waltham, USA) for visualization of corresponding size DNA bands.

### RT-qPCR efficiency and limit of detection evaluation

To compare RT-qPCR efficiency in different formats, a standard curve was constructed using either in vitro transcribed RNA or total RNA extracted from pool of serum/blood clinical samples from patients known to be positive for CASV, RIOMV, LNV and ANAJV hantavirus infection by RT-PCR and sequencing. Seven 1:10 dilutions of in vitro RNA were used for RT-qPCR amplification in two different formats: singleplex and duplex with MS2 EIC. In this study, we defined the LoD as the lowest amount of genome copies in a reaction that can be detected by the assays with 95% probability at a confidence interval (CI) of 95%. We also used endpoint sensitivity do determine LoD. Endpoint sensitivity is determined by the lowest amount of genome copies in a reaction where the target is not detected by the assay. First, we analyzed the LoD by defining the endpoint sensitivity of RT-qPCR and Semi-nested RT-PCR. For that, we tested 12 dilutions of viral RNA extracted from a pool made of samples from all four hantavirus strains previously quantified by RT-qPCR (genome copies ranging from 10^2^, 10^1^, 1 and 0.9 to 0.1 copies/μl in six replicates each). The LoD was thus defined as the lowest quantity that yield positive results in all six replicates. Next, we determined the 95% LoD for each strain individually. We used hantavirus RNA previously isolated from sylvatic rodent samples that were submitted for nucleotide sequencing. First, we quantified each stock RNA by RT-qPCR. Then we performed a serial 5-fold dilution starting at 100 copies/reaction down to 0.0062 copies/reaction per dilution for each strain. Each dilution series was tested six times in a single run and the results used to calculate the 95% LOD of the assay by probit regression analysis, using SPSS Statistics version 25 (IBM, USA). We then calculated the analytical sensitivity defined as amount of genome copies per reaction detected 95% of the time. In addition, we determined the LoD of RT-qPCR with Semi-nested RT-PCR by testing 12 dilutions of viral RNA quantified by RT-qPCR ranging from 10^2^, 10^1^, 1 and 0.9 to 0.1 copies/μl.

### Hantavirus ELISA assay

All positive samples used in this study were previous tested by ELISA assay for detection of IgM and IgG antibodies[54] at the Hantavirus Laboratory of Evandro Chagas Institute (Ananindeua, Brazil). For the detection of hantavirus antibodies, IgM and IgG ELISA assays were performed using the N recombinant antigen produced for the Andes virus[29] adapted to the Ksiazek protocol (CDC/Atlanta). Briefly, recombinant nucleocapsid protein was applied to the solid phase of a microtiter plate. After washing, serum samples were added. After 1-hour incubation at 37ºC plates were washed again and the conjugate (anti-human IgG) was used to detect immunoglobulins. The chromogenic substrate used was 2,2'-azino-di(3-ethyl-benzthiazoline sulfonate) (Kirkegaard and Perry Laboratories). Optical densities were read at 405 and 450 nm. Samples with indeterminate results were not included. Samples were considered positive for recent orthohantavirus infection when either IgM was detected in a single sample or when IgG was detected in two samples collected 15 days apart of each other with evidence of seroconversion.

### Clinical sensitivity and specificity

In order to access the RT-qPCR and Semi-nested RT-PCR assays clinical sensitivity and specificity for detection of orthohantavirus genome in human serum and blood samples, we performed a head to head comparison of each new assay with HPS IgM-ELISA assay. We chose IgM-ELISA as the reference test in our study because it is currently considered the gold standard for HPS diagnostic as viral isolation is often difficult and not used in diagnostic. We used a panel comprised of two different sets of samples (S1 Fig). The first set contains 50 samples collected between the years 2003 and 2012 from symptomatic patients. These are HPS positive samples previously confirmed by a retrospective IgM-ELISA testing. The second set contains another 50 samples collected from healthy individuals living outside endemic areas that agreed to participate in this study by signing a Consent form. These samples were then tested in this study by IgM-ELISA in order to confirm they were negative for HPS. All IgM-ELISA assays were performed by the Hantavirus Laboratory of Evandro Chagas Institute. Only samples that met the eligibility criteria (IgM-ELISA positive results and HPS suspected cases) were selected for the positive panel, and volunteers for the negative samples that met the eligibility criteria (healthy individuals with no risk of HPS exposal) were chosen at random. No clinical information from samples was available for the performers of IgM-ELISA, RT-qPCR or semi nested RT-PCR. The performers of all IgM-ELISA assays did not know the results for the index tests. Similarly, the index tests performers had access to the IgM-ELISA results only after the assays. The number of samples tested was determined by the number of confirmed HPS positive samples that were available with sufficient volume for testing from the HL collection (n = 50). Then, we collected another 50 confirmed HPS negative samples based on an artificial disease prevalence of 50% (50 positive samples out of 100 total samples tested). Another 27 samples (serum/blood, cell culture or mouse brain) infected with other pathogens (Table 2) were used to evaluate the assays analytical specificity. For RT-qPCR reactions, all samples were tested in triplicate and a No Template Control (NTC) as well as a Negative Control (NC) Sample and a Standard Curve were added to each run. For Semi-nested RT-PCR reactions was also included a Positive Control (PC). All results obtained by RT-qPCR were compared by those obtained by Semi-nested RT-PCR and ELISA. For the index tests (RT-qPCR and semi nested RT-PCR assays) there were only two possible outcomes were considered: positive or negative. For RT-qPCR, samples were considered positive if they crossed the CT cut-off value of < 40 in two or more replicates. Samples with indeterminate CT values or with CT >40 in at least two replicates were considered negative. For Semi-nested RT-PCR samples were considered positive if they presented an amplification band of approximately 241bp in an agarose gel. For the reference test (IgM-ELISA) we also considered only positive or negative outcomes. Samples were considered positive when either IgM was detected in a single sample or when IgG was detected in two samples collected 15 days apart of each other, by crossing the predefined cut-off value of > 0.2 O.D. All indeterminate results were excluded from the analysis. No missing data were observed for either the index tests or the reference test.

**Table 2:** Virus and other pathogens tested for specificity.

Pathogen	Sample type
*Dengue virus*, type 1	human blood
*Dengue virus*, type 2	human serum
*Dengue virus*, type 3	human blood
*Dengue virus*, type 4	human serum
*Yellow fever virus*, wild type strain H111	suckling mice brain
*Yellow fever virus*, vaccine strain 17DD	suckling mice brain
*West Nile virus*	suckling mice brain
*Saint Luis encephalitis virus*	cell culture
*Mayaro virus*	cell culture
*Western equine encephalitis virus*	cell culture
*Madariaga virus*	cell culture
*Mucambo virus*	cell culture
*Oropouche orthobunyavirus*	cell culture
*Brazilian mammarenavirus*	suckling mice brain
*Flexal mammarenavirus*	suckling mice brain
*Tacaribe mammarenavirus*	suckling mice brain
*Rabies lyssavirus*, antigenic variant 1	suckling mice brain
*Rabies lyssavirus*, antigenic variant 2	suckling mice brain
*Rabies lyssavirus*, antigenic variant 3	suckling mice brain
*Chikungunya* virus	suckling mice brain
*Rubella virus*	cell culture
*Ilheus virus*	cell culture
*Hepatitis C virus*	cell culture
*Plasmodium sp*.	human blood
*Leptospira sp*.	goat blood
*Paracoccidioides Lutzi*	yeast culture

### Statistical analysis

Statistical analysis was performed to define the assays diagnostic performance measures according to Ayres et al (2007)[57]. Chi-square and Kappa tests were used for association and concordance analysis, respectively. Kappa values vary from -1 (complete discordance) to 1 (complete concordance). Sensitivity, specificity, positive and negative predictive values and finally accuracy values were obtained by plotting the data into a contingency table containing the number of true or false positive and true or false negative results. Finally, a comparison was made between the results of each assay. All statistical analysis were performed in BioEstat software, version 5.3.

### Ethics statement

This study was approved by the Ethics Committee of Evandro Chagas Institute under the approval number: 371.953. All human subjects were adults and agreed to participate in this study by signing a written Form of Consent.

## Results

### RT-qPCR assay optimization

**Fig 1:**
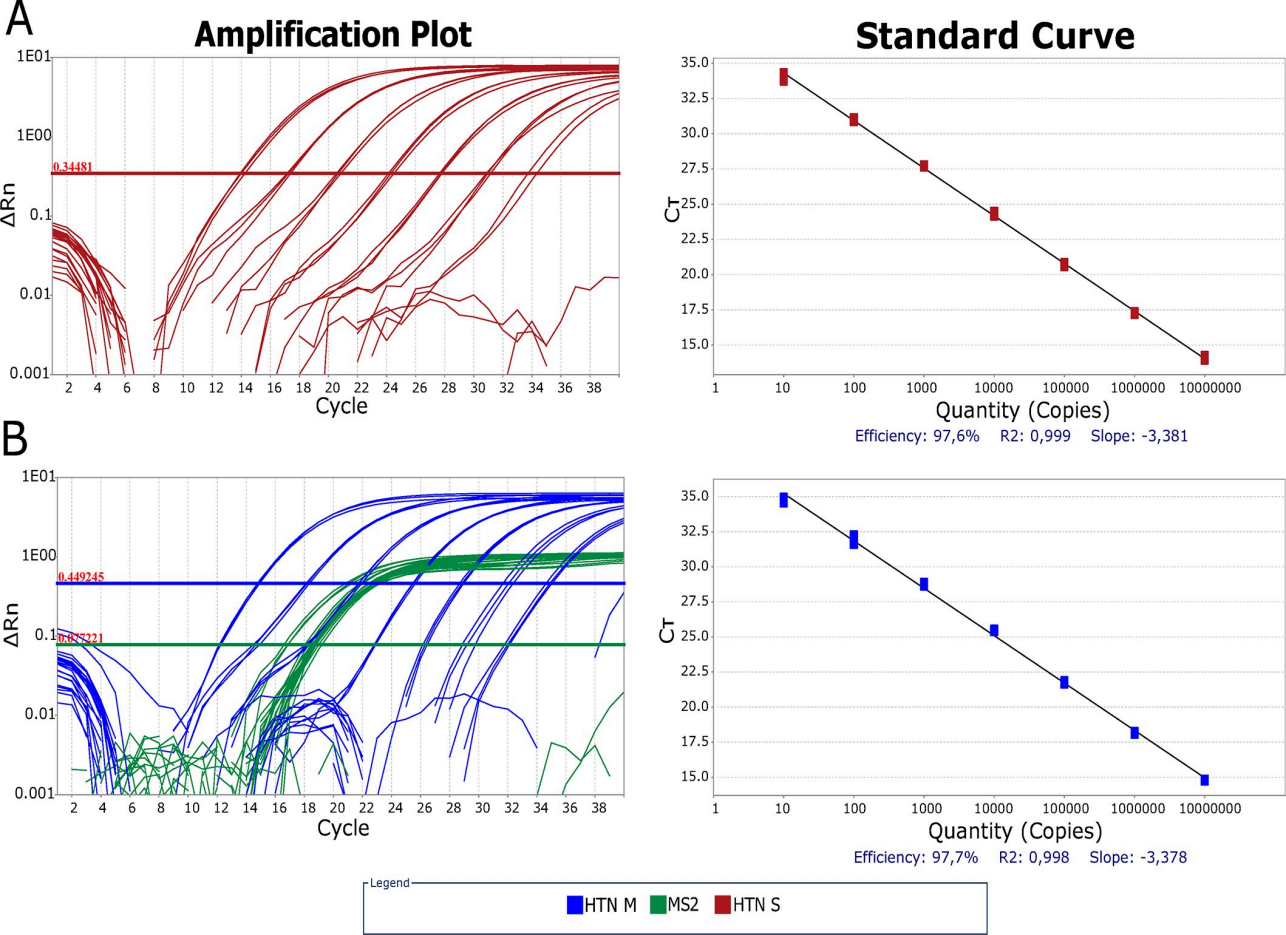
Comparison between amplification efficiencies of hantavirus RT-qPCR in singleplex and multiplex with MS2 EIC formats. Amplification plot and Standard Curve for each assay format are depicted. Threshold was fixed where the amplification efficiency was higher, inside the exponential phase of each amplification plot in logarithmic scale, with 0.3481 ΔRn in singleplex (A) and 0.4492 ΔRn in multiplex (B). Ct variation between singleplex and multiplex was within 1 Ct. Standard curves were constructed with seven 1:10 dilutions ranging from 10^7^ copies to 10^1^ copies of *in vitro* RNA. Amplification efficiency was 97.6% for singleplex (R^2^:0.999) (A) and 97.7% for multiplex (R^2^:0.998) (B). Ct: Cycle threshold, HTN M: hantavirus RT-qPCR multiplex format, HTN S: hantavirus RT-qPCR singleplex format.

The RT-qPCR assay developed in this study was able to amplify both in vitro RNA and viral genomes present in symptomatic patient samples. The primer/probe concentration that provided the best CT and ΔRn values were 900/250nM for hantavirus set and 100/50nM for EIC set. CT values obtained when using the two steps format were lower than those obtained when using the one-step format (mean CT values of 22.1 x 23.2). Two standard curves were tested to evaluate whether the assay would have the same efficiency in singleplex and duplex formats. We observed no difference in reaction efficiency and less than one Ct change between singleplex vs duplex format (Fig 1). The LoD determined by endpoint sensitivity of RT-qPCR assay was 9 copies/reaction or 180 viral genome equivalents per ml of blood (VGE/mL), whereas the limit of quantification (LOQ) was set in 10 copies/μL with R^2^ values varying between 0.98 to 0.99 in all dynamic linear range (DLR) up to five logs above the LoQ (10^7^−10^2^) (Fig 1). On the other hand, the Semi-nested RT-PCR was able to detect no less than 100 copies/reaction (Table 3). As for the individual 95% LoD for each strain, we found that the LoD for CASV and ANAJV (both ANDV strains) and RIOMV and LNV (both LNV strains) were 9.0, 5.8, 6.2 and 7.1, respectively, in copies per reaction (95% CI).

**Table 3:** Detection limit of hantavirus RT-qPCR and Semi-nested RT-PCR assays.

RNA copies/reaction	VGE/mL	RT-qPCR		Semi-nested RT-PCR
		positive/tested	Interpretation	
10^2^	2x10^3^	3/3	Positive	Positive
10	200	3/3	Positive	Negative
9	180	6/6	Positive	Negative
8	160	4/6	Negative	Negative
7	140	4/6	Negative	Negative
6	120	0/6	Negative	Negative
5	100	3/6	Negative	Negative
4	80	0/6	Negative	Negative
3	60	1/6	Negative	Negative
2	40	1/6	Negative	Negative
1	20	1/6	Negative	Negative
NC	NA	0/6	Negative	Negative
NTC	NA	0/6	Negative	Negative

VGE: viral genome equivalents; Ct: cycle threshold; NC: negative control; NTC: no template control; NA: not Applicable; Ind: indeterminate

### Clinical sensitivity and specificity tests

**Fig 2:**
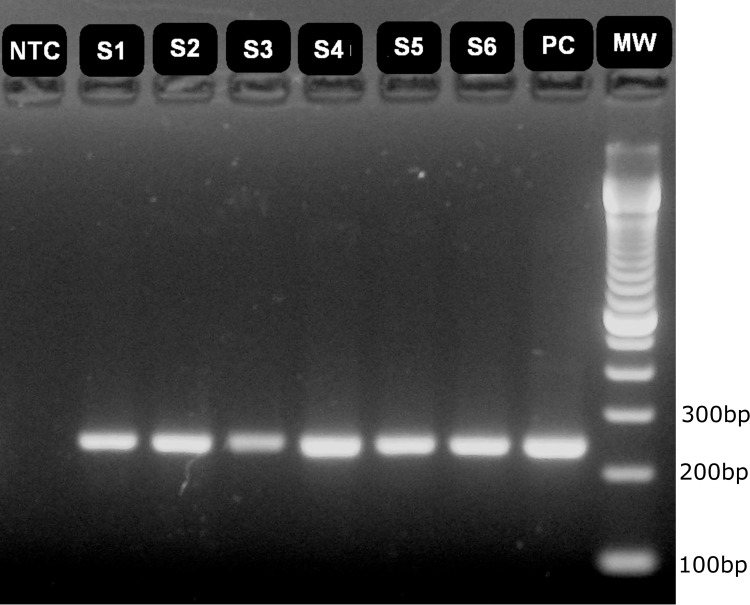
Agarose gel electrophoresis of hantavirus Semi-nested RT-PCR amplification products from clinical samples positive for IgM-ELISA. Agarose gel (1.5%) stained with SYBR safe dye were run for 50 min at 120V in TBE buffer. Each lane was loaded with 5μL of sample diluted in BlueJuice^™^ Gel Loading Buffer (Invitrogen). NTC: No template Control, S1-6: sample 1–6, PC: Positive Control, MW: Molecular weight (100bp DNA Ladder—Invitrogen).

A panel comprising of 127 samples was used to evaluate the assays clinical sensitivity and specificity. For RT-qPCR all samples that obtained CT values lower than 39 in all triplicates were considered positives results. All negatives results were validated by amplification of the EIC. Quantification of samples were given in copies/μL of RNA and only those quantification values within the assay LDR were considered, values bellow the LoQ were expressed as <10 copies/μL. It was not observed amplification of NTC or NC in any of the runs and reaction efficiency was in the usual acceptable level (varying from 95 to 105%) with R^2^ values ranging from 0,98–0,99. For Semi-nested RT-PCR, a sample was considered positive if its amplification produced a corresponding size band (241bp) in agarose gel (Fig 2). From the 50 clinical samples positive for IgM ELISA, 40 were positive for RT-qPCR and 37 for Semi-nested RT-PCR. Quantification of hantavirus RNA copies in samples varied from 1.2x10^6^ to 2.4x10^3^ copies/mL, with most of the samples (n = 25) falling below the LoQ (<2x10^3^ copies/mL). In order to further evaluate these samples which fell below the LoQ, we perform two additional experiments. First, we quantified total RNA from samples which had sufficient volume for RNA re-extraction (N = 16 out of 25). We used Qubit RNA BR Assay in the Qubit digital fluorimeter (Invitrogen, Thermo-Fisher Scientific, Waltham, USA). Of those 16 samples, only 6 were able to be quantified, with concentration ranging from 4.8 to 12.0 ng/μL. All the remaining samples presented less than 1 ng/μL (S3 Table). Of note, we did add exogenous MS2 RNA to each sample before extraction but only 0,1 ng per sample, which wouldn’t be detectable by the fluorimeter. Next, we tested these samples for three genes commonly used as endogenous internal control in RT-qPCR (GAPDH, β-actin and Rnase P) as well as for our EIC MS2. All 16 samples were positive for MS2 EIC, with CT values within the expected range (CT = 16–19), which indicates that the RNA extraction procedure worked adequately. However, only two samples were positive for RNase P gene and all were negative for both GAPDH and β-actin genes (S3 Table). Higher viral loads (>10^5^ copies/ml of blood) were detected in samples collected before five days of disease onset. Since there was no demographical data available from positive samples, we also did not collect demographical data from volunteers who donate the negative samples. Regarding the epidemiological information of patients, all data available as well as the assay results from the 50 IgM ELISA positive samples are shown in Table 4. The disease severity varied from absence of symptoms to death (Table 4). All 50 samples from healthy individuals as well as the 27 samples positive for other pathogens were negative for both assays and positive for the EIC MS2. All volunteers who donated the negative samples presented no clinical manifestations at the time of recruiting nor in the past 15 days before samples were collected. It should be mentioned, however, that no differential diagnostic was performed on those samples. Semi-nested RT-PCR assay presented clinical sensitivity and specificity of 74 and 100% respectively when compared with IgM ELISA (Table 5). On the other hand, RT-qPCR assay showed clinical sensitivity of 80% and specificity of 100% (Table 6). Positive and Negative predictive values as well as accuracy and kappa index values obtained by the comparison of the results obtained by Semi-nested RT-PCR with those obtained by RT-qPCR are shown in Table 7. Although index and reference tests were not carried out at the same time, they were performed on the same samples which were collected at the same moment during the disease progression of each patient. As such, there were no clinical interventions between the index test and the reference test, as they were performed on the same clinical samples. In addition, no adverse events were observed during the negative sample collection from volunteers. On the other hand, we have no information on adverse events that may have occurred during the positive samples collection from patients.

**Table 4:** Epidemiological data from hantavirus IgM-ELISA positive samples collected from symptomatic patients between 2003 and 2012 used in the clinical sensitivity/specificity panel.

#	DIAGNOSTIC ASSAYS RESULTS			EPIDEMIOLOGICAL DATA		
	IgG ELISA	Semi-nested RT-PCR	RT-qPCR Quantification (VGE/mL)	Disease time (days)	Clinical Manifestations	Year
01	Positive	Positive	<2x10^3^	2	NI	2006
02	Positive	Positive	<2x10^3^	3	NI	2006
03	Positive	Positive	<2x10^3^	6	NI	2006
04	Positive	Positive	3.8x10^5^	4	NI	2006
05	Negative	Positive	1.8x10^5^	4	NI	2006
06	Negative	Positive	<2x10^3^	NA	None (contact)	2006
07	Negative	Positive	1.6x10^5^	NI	Death	2006
08	Positive	Positive	3.8x10^4^	NI	NI	2006
09	Positive	Positive	8.1x10^5^	3	NI	2007
10	Positive	Positive	1.8x10^4^	1	Subclinical	2007
11	Positive	Positive	<2x10^3^	9	HPS	2007
12	Positive	Negative	Negative	8	NI	2008
13	Negative	Positive	<2x10^3^	6	Prodrome	2008
14	Negative	Negative	Negative	3	NI	2008
15	Negative	Negative	Negative	3	NI	2008
16	Negative	Negative	Negative	6	HPS	2008
17	Positive	Positive	1.1x10^4^	6	Prodrome	2008
18	Positive	Positive	<2x10^3^	5	HPS	2008
19	Positive	Negative	Negative	NI	NI	2006
20	Negative	Negative	<2x10^3^	4	Prodrome	2008
21	Negative	Negative	<2x10^3^	4	Prodrome	2008
22	Negative	Positive	<2x10^3^	3	Prodrome	2008
23	Negative	Negative	Negative	5	Death	2008
24	Positive	Positive	1.3x10^5^	NI	NI	2006
25	Positive	Negative	Negative	1	NI	2009
26	Positive	Negative	Negative	5	Neurologic	2009
27	Positive	Positive	<2x10^3^	7	NI	2009
28	Positive	Negative	Negative	2	NI	2009
29	Negative	Positive	1.2x10^6^	NI	NI	2009
30	Negative	Positive	<2x10^3^	6	NI	2007
31	Positive	Negative	Negative	11	NI	2009
32	Positive	Positive	3.5x10^5^	3	Death	2009
33	Positive	Positive	<2x10^3^	4	Prodrome	2009
34	Positive	Positive	<2x10^3^	5	NI	2010
35	Positive	Positive	<2x10^3^	NI	NI	2007
36	Positive	Positive	<2x10^3^	4	NI	2010
37	Positive	Positive	5.4x10^3^	7	NI	2010
38	Positive	Positive	<2x10^3^	3	NI	2010
39	Positive	Positive	1.0x10^5^	4	Prodrome	2010
40	Positive	Positive	9.0x10^4^	5	NI	2010
41	Positive	Positive	<2x10^3^	7	Prodrome	2010
42	Positive	Positive	2.4x10^3^	3	Prodrome	2010
43	Positive	Positive	<2x10^3^	NI	Death	2010
44	Positive	Positive	<2x10^3^	5	NI	2010
45	Negative	Positive	1.2x10^4^	NI	Prodrome	2008
46	Positive	Positive	<2x10^3^	6	Prodrome	2011
47	Negative	Negative	<2x10^3^	NI	NI	2008
48	Positive	Positive	<2x10^3^	NI	Death	2012
49	Positive	Positive	<2x10^3^	7	Death	2012
50	Positive	Positive	<2x10^3^	NA	None (contact)	2012

NA: not applicable; NI: not informed; HPS: hantavirus pulmonary syndrome. Prodrome: non-specific influenza-like symptoms stage; Subclinical: contact patients with no symptoms; Neurologic: patients with neurologic manifestations such as encephalitis and meningitis.

**Table 5:** Contingency table showing the results obtained by Semi-nested RT-PCR compared with those obtained by IgM-ELISA as reference assay.

IgM-ELISA				
		Positive	Negative	Total
Semi-nested RT-PCR	Positive	37	0	37
	Negative	13	50	63
	Total	50	50	100
Performance Measures		Value		κ index
Sensitivity		74.00%		0.74 (p<0.0001)*
Specificity		100.00%		
PPV		100.00%		
NPV		79.37%		
FP		0.00%		
FN		26.00%		
Accuracy		87.00%		

*95% Confidence interval. PPV: positive predictive value, NPV: negative predictive value, FP: False-positive, FN: False-negative.

**Table 6:** Contingency table showing the results obtained by RT-qPCR compared with those obtained by IgM-ELISA as reference assay.

IgM-ELISA				
		Positive	Negative	Total
RT-qPCR	Positive	40	0	40
	Negative	10	50	60
	Total	50	50	100
Performance Measures		Value		κ index
Sensitivity		80.00%		0.80 (p<0.0001)*
Specificity		100.00%		
PPV		100.00%		
NPV		83.33%		
FP		0.00%		
FN		20.00%		
Accuracy		90.00%		

*95% Confidence interval. PPV: positive predictive value, NPV: negative predictive value, FP: False-positive, FN: False-negative.

**Table 7:** Contingency table showing the results obtained by Semi-nested RT-PCR compared with those obtained by RT-qPCR as reference assay.

RT-qPCR				
		Positive	Negative	Total
Semi-nested RT-PCR	Positive	37	0	37
	Negative	3	87	90
	Total	40	87	127
Performance Measures		Value		κ index
Sensitivity		92.50%		0.94 (p<0.0001)*
Specificity		100.00%		
PPV		100.00%		
NPV		96.67%		
FP		0.00%		
FN		7.50%		
Accuracy		97.64%		

*95% Confidence interval. PPV: positive predictive value, NPV: negative predictive value, FP: False-positive, FN: False-negative.

## Discussion

HPS clinical diagnostic is usually difficult to be determined, especially in initial stages of disease due to the nonspecific nature of symptoms that mimetic many different febrile illnesses. As such, one should always take into consideration the patient epidemiological information in association with laboratory test results to make a safe conclusive diagnostic[58]. Routine laboratory diagnostics of suspected cases is often based solely in antibodies detection by ELISA without looking at patient viremia. One of the drawbacks of serology is the need to investigate seroconversion by testing a new sample collected two to three weeks after the first one, when the first serum is considered inconclusive. However, in order to evaluate whether the assays developed here would help to elucidate ELISA ambiguous results, a set of inconclusive samples should be tested. In most severe cases, however, patients clinical condition worsen dramatically and many of them die even before the time when antibodies are detectable[59], which makes quick diagnostic elucidation very important as any delay may have great impact in patients evolution. Accordingly, molecular biology techniques may be used in parallel to serology for early detection of viral genome, really speeding up results. Due mainly for its high sensitivity and specificity, RT-PCR have been commonly used not only in hantavirus diagnostic but for many other viral pathogens as well[37].

One of the challenges for hantavirus molecular detection lies in the notably nucleotide diversity observed between different virus associated with different reservoirs[23,31,60–63]. As a result, most of RT-qPCR protocols for hantavirus are developed targeting specific species or group of species that circulate at predefined regions[39,51–53,64]. For example, there is only one RT-qPCR protocol available for detection and quantification of the Brazilian hantaviruses ARAV and RIOMV[53].

Genomic S segment from Amazon hantaviruses was chosen as target for both RT-qPCR and Semi-nested RT-PCR protocols. The N gene is relatively conserved among hantaviruses and is often used as template to design primers and probes for these viruses[31,33,65,34,39–42,52,53,64]. Hydrolysis TaqMan probes were used instead of SYBR Green as this system allows for the detection of more than one target in the same reaction. In addition, the TaqMan system has been used more often in the diagnosis of infectious diseases, mainly due to its higher specificity and with easy standardization, with no need to perform dissociation curves[66].

MS2 bacteriophage RNA was used as exogenous IC for the RT-qPCR reactions in this study. The use of MS2 as IC was first suggested by Dreier et al (2005) and since then, it has been reported in other studies[55,67]. MS2 RNA was used as a non-competitive IC as competitive ICs may decrease amplification efficiency of the target which may lead to lower detection limits[68].

Some studies reported the use of Nested RT-PCR technique for hantavirus genome detection[31,32,34,35]. Most of them are based on the protocol developed by Nichol et al. (1993) which uses in the first amplification step a pair of primers targeting a conserved sequence of an Old World hantavirus, and in the second step (Nested) a pair of primers against sequences obtained from hantaviruses circulating in the USA[30]. Moreli et al. (2004) developed a nested RT-PCR protocol for hantaviruses from South America based on sequences from ANDV, RIOMV, ARAV and CASV. The primers designed in this study, on the other hand, were based on sequences from hantavirus circulating in the Amazon region[23].

For standard curve construction we used in vitro transcribed RNA instead of plasmid DNA as DNA amplification efficiency do not always correlates with that from RNA[69]. Several protocols for quantification of orthohantavirus and other RNA viruses also use RNA standard curves[44,47,53,64,70–74]. We also verified if there was any difference in amplification efficiency of viral genome and in vitro transcribed RNA. We found that the efficiency values ​​obtained in standard curves performed with each RNA were very close, which indicates that amplification of in vitro RNA corresponds to hantavirus genome amplification.

Optimal primers and probe concentration was determined by evaluating different concentration combinations. Typically, primer concentration in a qPCR reaction may range from 50-1000nM, while probe concentration may range from 50-300nM. A high primer concentration may increase detection sensitivity but may also decrease specificity by favoring non-specific amplifications. Although the concentration used here was close to the maximum limit recommended, it provided early detection of the target, with lower Ct value when compared to the other concentrations tested. Importantly, this high concentration did not impair reaction specificity as nonspecific amplifications were not observed. To prevent EIC from interfering with hantavirus RNA amplification, EIC primers and probe concentration had to be decreased to its limiting concentration. In order to achieve this, several concentrations of MS2 primers and probes were tested. After changing to the limiting concentration, efficiency values ​for hantavirus RNA amplification were very similar in both singleplex and multiplex format.

LoD and LoQ for qPCR methods can be estimated from analysis of replicate standard curves. From the definition of LoD follows that working at 95% confidence, LoD is the measured concentration that produces at least 95% positive replicates[75]. Our RT-qPCR protocol was able to detect at least 0.9 copies/μL of orthohantavirus RNA in 100% of the replicates (6 out of 6), thus showing excellent analytical sensitivity with lower LoD when compared to other studies that reported LoDs varying between 2 to 1000 copies/μL of RNA[39–42,47,51,53,73]. In addition, the assay was able to accurately quantify from 10^1^ to 10^7^ copies/μL of RNA, with linearity coefficient varying between 0.98–0.99, calculated from the standard deviation (SD) of CT values obtained in the standard curve (Fig 1). This result showed that we were not able to precisely quantify less than 100 copies/reaction of hantavirus RNA, despite still being capable of detecting 9 copies/reaction of viral genome. This is an expected limitation of the technique since amplification linearity decreases dramatically as it approaches its LoD, which often impairs accurate quantification of low target amounts[76,77]. For quantification of those scarce targets, the ddPCR (digital droplet PCR) technique is usually better indicated due to its greater sensitivity[78] and higher overall metrological quality[79,80], especially with low target amounts[81–83]. Unfortunately, we were not able to isolate CASV, LNV, RIOMV and ANAJV, as hantavirus isolation is often difficult^55^. Although we have designed our assays to target a very conserved genome region from South American hantaviruses, more tests are needed in order to evaluate if there is any difference in the assays efficiency and sensitivity in respect of the amplification of each of these strains individually. Similarly, we also couldn’t correlate un-quantifiable viremia or discrepancy between RT-qPCR and IgM ELISA by strain.

Next, we compared the analytical sensitivity between the RT-qPCR and the Semi-nested RT-PCR assays by testing viral RNA Standards of known concentration by both methods. RT-qPCR was about 10-fold more sensitive than Semi-nested RT-PCR. This result shows how the sensitivity of the Semi-nested RT-PCR is close to the RT-qPCR, which is expected since the nested step was developed precisely to increase detection sensitivity over the conventional PCR assay. Morelli et al (2004) showed 100% positivity for the nested RT-PCR compared to 88.9% positivity for the conventional RT-PCR. Several other studies have compared the sensitivity of qPCR and nested PCR methodologies. Some of these data is in accordance with our results, with qPCR more sensitive than the nested PCR[40,41,53,64,84]. However, other studies have reported comparable sensitivities between both methods[42] or even nested PCR presenting a greater sensitivity than qPCR[85,86]. Therefore, the difference of sensitivity levels between the two methodologies seems to vary from protocol to protocol[87]. We also defined the 95% LoD for each hantavirus strain individually, in order to evaluate whether there is any difference in sensitivity in the detection each specific strain. We found that the LoD for CASV and ANAJV (both ANDV strains) and RIOMV and LNV (both LNV strains) were 9.0, 5.8, 6.2 and 7.1, respectively, in copies per reaction (95% CI). Based on this data, we may assume that there is no expressive difference between the LoD of the assay related to the different strains tested, with all four falling within the same Log of copies/reaction. In addition, we accessed the assays analytical specificity by testing 27 samples positive for other pathogens that may cause similar disease profile. All serum/blood samples (Table 2) were obtained from naturally infected patients with high viremia confirmed by RT-qPCR (CT values ranging from 15–20). Similarly, RNA extracted from cell culture and suckling mice brain samples (Table 2) was tested for the presence of their respective pathogens RNA by RT-qPCR. The viral load in this sample type is usually much higher than the physiologic concentrations of those pathogens in natural infections, as we observed in the RT-qPCR CT values (CT < 10). Even so, all samples were negative for both assays, showing that there was no cross amplification of these pathogens RNA by the primer/probe sets designed in this study, as it was expected by in silica analysis.

The protocols performance with clinical specimens was evaluated using a panel containing 100 serum/blood samples: 50 IgM-ELISA positive and 50 IgM/IgG-ELISA hantaviruses negative samples. All 50 IgM/IgG ELISA negative samples from healthy subjects were negative for both RT-qPCR and Semi-nested RT-PCR, indicating that both protocols are specific. Among the samples positive for IgM-ELISA, RT-qPCR obtained a positivity of 80% (40/50) whereas Semi-nested RT-PCR had positivity of 74% (37/50). These data confirm the lower analytical sensitivity observed for the Semi-nested RT-PCR and corroborates what have been shown in other studies. Jiang et al. (2014) reported a 100% (27/27) positivity of serum positive samples IgG-ELISA whereas Machado et al. (2013) observed a 50% (10/20) positivity of IgG-ELISA serum samples positive for RT-qPCR and 40% (8/20) for conventional RT-PCR. Evander et al (2007) found a positivity of 78.4% (40/51) of IgM-ELISA positive serum samples in both RT-qPCR and Semi-nested RT-PCR. Ten samples positive for IgM-ELISA were negative for RT-qPCR. Most of them (6/10) are also positive for IgG-ELISA and all of them are old samples collected before 2009. Studies have shown that there is an inversely proportional correlation between IgG antibody levels and hantavirus viral load[42,43,53]. It is possible that a low viral load, below LoD, due to high titers of IgG antibodies together with loss of RNA quality and quantity from prolonged storage time and repeated thawing and freeze cycles may have impaired the results for these samples. For a complete diagnostic validation, however, these assays should be submitted to a blind head to head comparison between PCR and IgM ELISA in a diagnostic routine using all sorts of suspected HPS samples, including not only samples with ambiguous results but also HPS suspected samples with negative IgM ELISA results (which also wasn't tested in this study). Determination of the number of samples should take into consideration the real disease prevalence of HPS in the region. Is important to remind that all positive samples used here were stored for a long time and were probably submitted to repeated cycles of freezing and thawing for diagnostic confirmation whereas the negative samples were freshly collected and only thawed once. This may represent a potential source of bias in our analysis. In addition, we used groups of six replicates in order to determine the assays LoD. A larger number of replicates is recommended in order to reduce the statistical uncertainty in the definition of the assays LoD.

Regarding general accuracy, both methods obtained very close values, with RT-qPCR scoring slightly higher accuracy (90% vs. 87%), since the Semi-nested RT-PCR failed to detect three samples. RT-qPCR also showed excellent correlation with IgM-ELISA with a kappa number of 0.8 (p <0.0001), whereas Semi-nested RT-PCR showed a good correlation with kappa number of 0,74 (p <0.0001). However, the best correlation was obtained when comparing the two methods with each other, with a kappa index of 0.94 (p <0.0001), which is also expected since both methods detect viral genome. It is important to note, however, that samples with artificial disease prevalence (in this case, 50%) tend to either overestimate or underestimate positive predictive value measures if they are higher or lower than the real disease prevalence in practice, respectively.

Between the 40 samples positive for RT-qPCR, more than half (25/40) fell below the LoQ of 10 copies/μL of RNA, and therefore its quantification was not considered. Interestingly, most of them (19/25) were also IgG positive and the remaining were stored more than 6 years and were submitted to repeated freeze and thaw cycles for diagnostic confirmation. Additionally, we investigated whether these low quantification samples were indeed due to low viral genome copy loads or if there were low amounts of total RNA in samples due to either inefficient RNA extraction or poor RNA quality. We observed that total RNA concentrations of these samples are well below the expected values. In addition, only 3/16 samples had positive signals by RT-qPCR for 1/3 housekeeping genes even though all 16 were positive for MS2 EIC. These data suggest that RNA quantity and perhaps even RNA quality was poor in the original sample. In this context, severe damaged RNA may have impaired hantavirus genome quantification for these samples. As we mentioned previously, all the HPS positive samples we used were obtained from HL of IEC, where they may have been submitted to several freeze and thaw cycles for HPS and differential diagnostics in other labs of IEC. This probably had a significant impact in viral load of these samples, especially those with low viremia to begin with. Quantification of the other samples ranged from low viral loads such as 2.4 x 10^3^ copies/mL to concentrations as high as 1.2 x 10^6^ copies/mL. These data were similar to those obtained by Evander et al (2007), with a PUUV viral load ranging from 1.7x10^2^–3.0x10^6^ copies/mL; by Xiao et al. (2006) which obtained SNV viral loads of 1.7x10^4^ to 1.8x10^6^ copies/ml and by Machado et al. (2013), which found RIOMV and ARAV viral loads of 6.64 x 10^4^–3.78 x 10^6^ copies/ml. Other studies, however, reported viral loads as high as 10^7^ copies/mL for HTNV[64], 10^8^ copies/μL for DOBV[43] and 10^9^ copies/mL for SNV[36]. It is important to point out that the highest loads quantified in this study were from samples obtained from patients with less than five days since disease onset, including two fatal cases. These results are in agreement with literature data that shows highest viral loads in patients with less than five days of disease[42,50].

Clinical specificity and sensitivity of new diagnostic tools are defined by comparing its results with those obtained by a reference or gold standard method. Here we considered IgM-ELISA as the gold standard because it is the most widely used method for the diagnosis of hantavirus. Although ELISA is a serological method, hantavirus infections shows a slightly different antibody production kinetics, with detectable IgM antibodies as early as the first days of disease onset[88,89]. This favors the simultaneous detection of viral RNA and antibodies and allows many studies to compare molecular methods with serological methods for detection of hantavirus[42,64]. In addition, hantaviruses are notably difficult to grow in cell culture[59,62,90], and virus isolation was not possible in this study.

Taken together, these results indicate that both RT-qPCR and Semi-nested RT-PCR assays are efficient, sensitive and specific tools for genome detection of hantaviruses circulating in the Amazon region. In addition to being more sensitive, RT-qPCR offers other advantages such as agility in generating results, about 3 times faster than conventional assays and automation capability which leads to lower risk of contamination and greater reproducibility. Furthermore, the possibility of absolute quantification of the viral RNA load present in the sample may be an important tool that could clarify aspects about the relationships between viral load, pathogenesis, virulence and immune response in hantavirus infection. Differently from other virus infections, the antibody production kinetics in HPS allows the detection of IgM and even IgG early in the disease, which is one of the main reasons why ELISA has been widely used in HPS diagnostic[91]. The use of RT-qPCR in association with ELISA in HPS diagnostics may be beneficial as nucleic acid detection is not as susceptible for cross-reactivity as it has been reported for ELISA antibody detection[54].

HPS represents an important public health concern in the Americas, with more than two thousand cases reported solely in Brazil, two thirds of that in the Amazon. In recent years we have witnessed an increase in deforestation, especially in the Amazon region, driven by industrial activities and large-scale agriculture. Which eventually leads to the replacement of the natural habitat of sylvatic rodents from a forest setting, where food is scarce, to an agricultural setting where crops of grains serve as a new source of food. This agricultural profile, with large areas of crops, favoring the maintenance of large populations of wild rodents, is a determining factor for hantavirus infection, because when the food supplied by these crops runs out periodically, the rodents segregated by competition leave in search of food in the residences, bins and surrounding silos^92^. In addition, areas deforested for construction of precarious human dwellings, where food are stored for their own consumption as well as to feed their domestic animals, also favors the contact of humans with rodents^92, 93^. Considering these factors, it wouldn’t be a surprise if the number of HPS cases increases in the next few years in the Amazon region following the close proximity between susceptible individuals and sylvatic rodent reservoirs favored by deforestation activities. In this sense, the assays developed in this study may become an important tool for rapid identification of hantavirus outbreaks, contributing with a sensitive and specific diagnosis for hantaviruses previously identified in the Brazilian Amazon as an add-on test in conjunction with IgM-ELISA.

## Supporting information

S1 Table Primer concentrations tested and the Ct e ΔRn values obtained. (DOCX)

S2 Table Primer and probe concentration combination tested and the Ct e ΔRn values obtained. (DOCX)

S3 Table Quantification of total RNA from positive samples that fell below the RT-qPCR LoQ. (DOCX)

S1 Fig Flowchart of participant flow used in this study design. (DOCX)
