# Supplementary material for: Development of RT-qPCR and semi-nested RT-PCR assays for molecular diagnosis of hantavirus pulmonary syndrome
Source: PLoS Negl Trop Dis. 2019 Dec 26;13(12):e0007884. doi: 10.1371/journal.pntd.0007884 (PMC6932758; doi:10.1371/journal.pntd.0007884)
Supplement: S1 Table — (DOCX) [file pntd.0007884.s001.docx]

**S1 Table**. Primer concentrations tested and the Ct e ΔRn values obtained

| **Final Concentration (nM)** | **Mean Ct** | **Mean ΔRn** | **Mean Ct NTC** | **Mean Ct CN** |
| --- | --- | --- | --- | --- |
| **900** | 16.273 | 5.743 | Indeterminate | Indeterminate |
| **700** | 16.653 | 5.612 | Indeterminate | Indeterminate |
| **500** | 17.209 | 5.186 | Indeterminate | Indeterminate |
| **300** | 18.067 | 4.384 | Indeterminate | Indeterminate |
| **100** | 20.334 | 2.035 | Indeterminate | Indeterminate |
| **50** | 22.334 | 1.133 | Indeterminate | Indeterminate |

RNA and probe concentration were 250nM e 10^5^ copies/µL, respectively.
