# Supplementary material for: Development of RT-qPCR and semi-nested RT-PCR assays for molecular diagnosis of hantavirus pulmonary syndrome
Source: PLoS Negl Trop Dis. 2019 Dec 26;13(12):e0007884. doi: 10.1371/journal.pntd.0007884 (PMC6932758; doi:10.1371/journal.pntd.0007884)
Supplement: S2 Table — (DOCX) [file pntd.0007884.s002.docx]

**S2 Table.** Primer and probe concentration combination tested and the Ct e ΔRn values obtained

| **Final Concentration Primers/Probe (nM)** | **Mean Ct** | **Mean ΔRn** | **Mean Ct NTC** | **Mean Ct CN** |
| --- | --- | --- | --- | --- |
| **900/250** | 16.270 | 5.362 | Indeterminate | Indeterminate |
| **900/200** | 16.687 | 3.538 | Indeterminate | Indeterminate |
| **900/150** | 16.472 | 2.794 | Indeterminate | Indeterminate |
| **900/100** | 16.820 | 1.931 | Indeterminate | Indeterminate |
| **900/50** | 17.363 | 0.881 | Indeterminate | Indeterminate |
| **700/250** | 17.170 | 4.383 | Indeterminate | Indeterminate |
| **700/200** | 17.002 | 3.888 | Indeterminate | Indeterminate |
| **700/150** | 17.149 | 2.913 | Indeterminate | Indeterminate |
| **700/100** | 17.098 | 2.007 | Indeterminate | Indeterminate |
| **700/50** | 17.824 | 0.872 | Indeterminate | Indeterminate |

RNA concentration was 10^5^ copies/µL
