# Supplementary material for: Development of RT-qPCR and semi-nested RT-PCR assays for molecular diagnosis of hantavirus pulmonary syndrome
Source: PLoS Negl Trop Dis. 2019 Dec 26;13(12):e0007884. doi: 10.1371/journal.pntd.0007884 (PMC6932758; doi:10.1371/journal.pntd.0007884)
Supplement: S3 Table — (DOCX) [file pntd.0007884.s003.docx]

**S3 Table.** Quantification of total RNA from positive samples that fell below the RT-qPCR LoQ

| **Sample #** | **Total RNA (ng/uL)** | | **MS2 EIC Ct values** | | **GAPDH Ct values** | | **Rnase P Ct values** | | **β-Actin Ct values** | |
| --- | --- | --- | --- | --- | --- | --- | --- | --- | --- | --- |
| 1 | 7.72 | 16.2 | | UND | | UND | | UND | |  |
| 2 | <1 | 16.5 | | UND | | UND | | UND | |  |
| 4 | 9.32 | 17.3 | | UND | | UND | | UND | |  |
| 7 | <1 | 16.2 | | UND | | UND | | UND | |  |
| 14 | 11.00 | 18.1 | | UND | | 34.1 | | UND | |  |
| 16 | <1 | 16.3 | | UND | | UND | | UND | |  |
| 26 | <1 | 16.5 | | UND | | UND | | UND | |  |
| 31 | <1 | 17.6 | | UND | | UND | | UND | |  |
| 34 | <1 | 16.2 | | UND | | UND | | UND | |  |
| 38 | <1 | 16.3 | | UND | | UND | | UND | |  |
| 39 | 12.00 | 19.2 | | UND | | 36.2 | | UND | |  |
| 40 | 4.80 | 16.0 | | UND | | UND | | UND | |  |
| 42 | 5.72 | 17.5 | | UND | | UND | | UND | |  |
| 47 | <1 | 16.4 | | UND | | UND | | UND | |  |
| 48 | <1 | 16.8 | | UND | | UND | | UND | |  |
| 55 | <1 | 16.3 | | UND | | UND | | UND | |  |

IND = Undetermined
