# Supplementary figures and images for: Development of RT-qPCR and semi-nested RT-PCR assays for molecular diagnosis of hantavirus pulmonary syndrome
Source: PLoS Negl Trop Dis. 2019 Dec 26;13(12):e0007884. doi: 10.1371/journal.pntd.0007884 (PMC6932758; doi:10.1371/journal.pntd.0007884)

**S1 Figure.** Flowchart of participant flow used in this study design


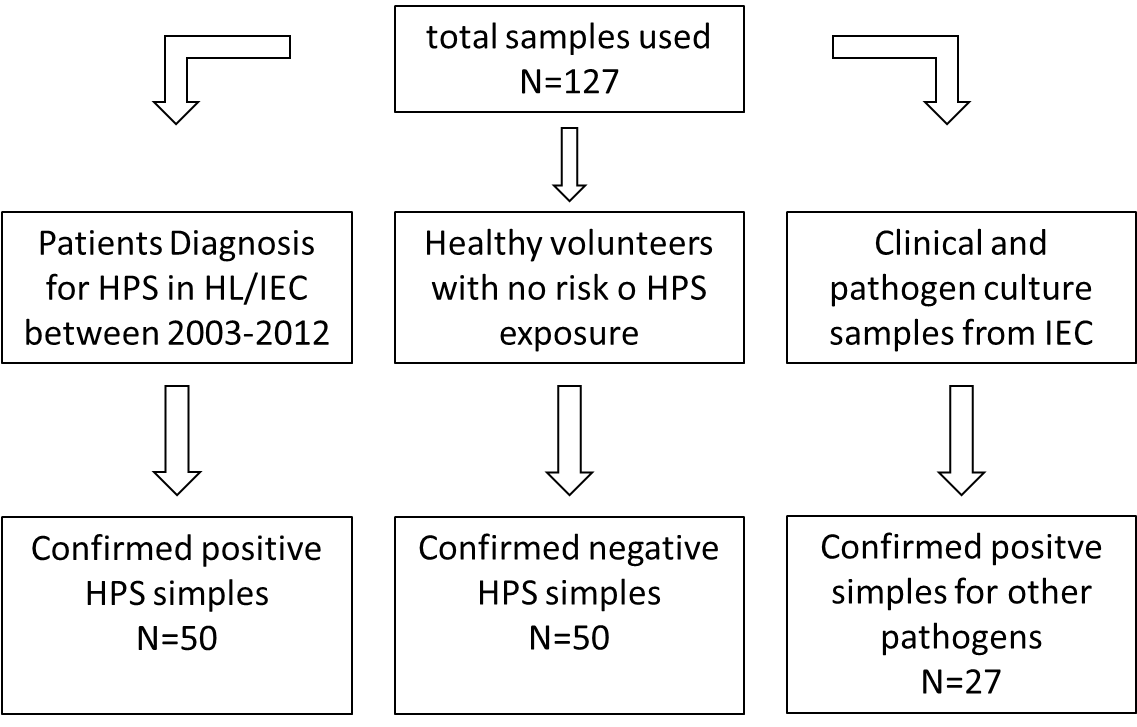

Supplement: S1 Fig — (DOCX) [file pntd.0007884.s004.docx]
